# Safety of fluralaner oral solution, a novel systemic antiparasitic treatment for chickens, in laying hens after oral administration *via* drinking water

**DOI:** 10.1186/s13071-017-2291-5

**Published:** 2017-08-08

**Authors:** Angella Prohaczik, Monika Menge, Bruno Huyghe, Annie Flochlay-Sigognault, Gaëlle Le Traon

**Affiliations:** 1 MSD Animal Health Innovation SAS, 7 Rue O. de Serres CS 67131, Beaucouzé Cedex, 49071 France; 2 MSD Animal Health Innovation GmbH, Zur Propstei, 55270 Schwabenheim, Germany; 3 Merck Animal Health, 2 Giralda Farms, Madison, NJ 07940 USA

**Keywords:** Fluralaner, Drinking water, Laying hen, Poultry, Chicken, Safety, Parasiticide, Isoxazoline, Poultry red mites

## Abstract

**Background:**

Poultry mites are the most significant pest affecting production systems in the egg-laying industry. Fluralaner is a novel systemic insecticide and acaricide that is effective against poultry mites (*Dermanyssus gallinae*, *Ornithonyssus sylviarum*) in chickens after oral administration. This study investigated the safety of oral administration of a 1% solution of fluralaner in drinking water to laying hens at the recommended treatment dose and at multiples of this dose.

**Methods:**

One hundred-twenty healthy 28-week-old laying hens, weighing 1.4–2.1 kg at first administration, were included in the study, and allocated to 4 treatment groups of 30 hens each receiving daily doses of 0, 0.5, 1.5 and 2.5 mg fluralaner/kg body weight, equivalent to 0, 1, 3, and 5 times the recommended dose of fluralaner. The product was administered *via* drinking water on a total of six occasions, as 3-day treatment periods twice with an interval of 4 days with no treatment (treatment on days 1, 2, 3 and 8, 9, 10), representing 3 times the recommended number of administrations. Hens supplied with non-medicated drinking water served as controls.

During the study, all hens were clinically observed, and their health was carefully monitored including body weight, food and water consumption, hematology, clinical chemistry, and withdrawal reflex test. Eggs laid over the study were evaluated for main characteristics (e.g. weight, shape, strength, shell thickness and soundness, albumen height, yolk color, Haugh unit and presence of blood and/or meat spots). Following euthanasia of the hens at the end of the second treatment period (day 11) or 18 days later (day 29), complete gross *post-mortem* examination, including organ weight determination, and histopathological examination of multiple tissues were conducted.

**Results:**

There were no clinical findings related to fluralaner treatment. Statistically significant differences between the treated groups and the control group were observed for some clinical pathology parameters; none of these findings were considered to be of clinical nor zootechnical relevance. Organ weights, gross post mortem and histopathological examinations did not reveal any finding associated with treatment with fluralaner.

**Conclusions:**

Oral administration of fluralaner *via* drinking water at the recommended treatment dose (0.5 mg/kg body weight twice at 1-week interval), is well tolerated and has a high safety margin up to an overall dose of 15 times the recommended one (5 times the daily dose given 3 times the number of days) in healthy adult laying hens. Based on the present results, the use of the new mite treatment based on fluralaner administered *via* drinking water is expected to be safe for laying hens under industrial conditions, and to have no negative impact on their egg quality and production.

## Background

Poultry mites are the most significant pest affecting production systems in the egg-laying industry. Few products are licensed for use against mites in layers. Fluralaner is a novel systemically administered insecticidal and acaricidal compound with efficacy against ticks and fleas and a demonstrated high margin of safety after oral administration to dogs [1]. A high efficacy of fluralaner was also shown against *Dermanyssus gallinae* [2], commonly named poultry red mite, a blood-sucking ectoparasite widely present in most of the laying hen facilities, with significant negative impact on bird health and production performances [3]. Treatment of poultry *via* the oral route is considered as a potential treatment to control red mite populations in the poultry buildings. Fluralaner is a potent inhibitor of ligand-gated chloride channels (γ-aminobutyric acid (GABA)- and L-glutamate gated chloride channels) in neurons with significant selectivity for arthropod neurons over mammalian neurons [2, 4, 5]. The oral safety of fluralaner has been investigated in mammals, including dogs and cats [1, 6], but no data are available on its safety in any avian species, particularly in food producing animals like layers, for which any possible health impact from treatment may subsequently decrease egg production and reduce economic performances of the treated flock. This study was designed to demonstrate the safety of this new systemic treatment and to investigate any possible health impact from repeated oral administration to healthy laying hens of multiple overdoses.

## Methods

### Subjects

This randomized, parallel-group, blinded study included 120 healthy 28-week old Novogen laying hens. A total of 90 hens received fluralaner and 30 untreated hens served as controls. The study design was based on VICH GL 43 target animal safety requirements for veterinary pharmaceutical products [7]. The study was conducted in accordance with the OECD Principles of Good Laboratory Practice (GLP).

Laying hens were enrolled in the study at 24 weeks of age (start of acclimation) and were confirmed as healthy based on clinical health observations, egg laying record and body weight record. Hens were housed in climate monitored rooms (12–24 °C) with a day length of 16 h light and 8 h darkness suitable for egg production. Hens were fed a standard commercial diet meeting the recommendations of the National Research Council [8] at recommended rates, and had access to drinking water ad libitum. Hens were housed in individual pens from day -21/-22 to the end of the study. No treatment other than the experimental one was administered to hens during the entire study.

### Allocation to treatment groups

**Fig. 1:**
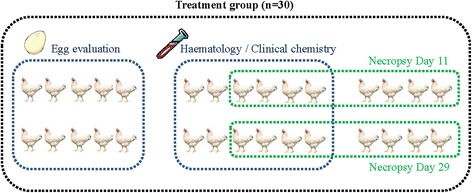
Allocation of hens within each treatment group to experimental subgroups dedicated to specific evaluations

Hens/pens were randomly allocated into treatment groups on day -14/-15 using a block randomization procedure. Thirty blocks of pens were formed based on the facility diagram such as 4 adjacent pens formed a block. Birds were grouped (4 to a group) based on similar body weight as measured on day -16/-17 and randomly assigned to block and pen within a block. Within each block, birds were randomly assigned to one of the 4 treatment groups. In addition, within each treatment group, 12 out of the 30 hens were randomly allocated to blood sampling for the analysis of haematology and clinical chemistry parameters. Finally, within each treatment group, 16 out of the 30 hens (8 blood sampled birds and 8 non-blood sampled birds) were randomly allocated to necropsy. Half of the birds were necropsied on day 11, and half of the birds on day 29. The 10 birds not blood-sampled and not allocated to necropsy were assigned to egg evaluation until day 36. This allocation process is summarized in Fig. 1.

### Treatments

The study was divided into four distinct phases: pre-treatment phase from randomization to first day of treatment on day 1 (*n* = 30 per group), main study phase from day 1 to day 11 (*n* = 30 per group), recovery phase 1 from day 11 to day 29 (*n* = 22 per group), and recovery phase 2 from day 29 to day 36 (*n* = 14 per group).

Three treatment groups received fluralaner at different doses and one group served as non-medicated control. The recommended dose rate of fluralaner in chickens is 0.5 mg/kg, to be administered twice at 7 days interval [9]. This study evaluated the oral administration of fluralaner, formulated as a 10 mg/ml solution, diluted into drinking water, at 1, 3, or 5 times the recommended treatment dose, i.e. at 0.5 (1× group), 1.5 (3× group), or 2.5 (5× group) mg fluralaner/kg body weight for a total of 6 administrations. Hens were administered fluralaner 6 times on days 1, 2, 3, 8, 9 and 10, with the first dose administered at *c.*28 weeks of age and 1.4–2.1 kg of body weight (Table 1). The hens were weighed before each 3-day period of treatment (day -1 and day 7) to calculate the appropriate fluralaner concentration in drinking water to reach each target dose rate. The target concentration of fluralaner in drinking water was calculated for each treatment group based on (i) the target dose rate for the group, (ii) its total body weight, and (iii) its total daily water consumption as estimated from individual daily water consumptions measured over the 5 days prior to each 3-day period of treatment (from day -6 to day -2, and from day 2 to day 6). On each treatment day, the appropriate amount of fluralaner from the 10 mg/ml solution of fluralaner was diluted into drinking water to reach the target concentration. The actual concentrations of fluralaner in medicated water were determined using a validated fast liquid chromatographic (LC) method with ultraviolet (UV) detection. Confirmatory analyses of the batches of medicated water prepared were conducted and the actual concentrations were used to determine the actual dose rates of fluralaner actually administered to the hens in the different treatment groups (Table 2). On each administration day, the amount of medicated water provided to each hen was weighed, as well as the amount remaining after 24 h, to determine the amount of medicated water actually ingested by each hen. The hens from the control group (0× group) received non-medicated tap water. Analysis confirmed that no fluralaner was detected in any of the batches of water provided to the hens from the control group.

**Table 1:** Hens body weights (kg) at the time of first treatment administration

Group	Control	Fluralaner 1×	Fluralaner 3×	Fluralaner 5×
Range	1.56–2.02	1.45–2.12	1.56–2.05	1.44–2.03
Mean	1.77	1.78	1.77	1.76

**Table 2:** Fluralaner dose [mean (range)] administered to hens in each treatment group

Treatment	Dose range per group (mg/kg)		
	Fluralaner 1×	Fluralaner 3×	Fluralaner 5×
First administration (day 1)	0.48 (0.35–0.85)	1.51 (1.14–2.04)	2.39 (1.56–3.37)
Second administration (day 2)	0.50 (0.30–0.70)	1.50 (1.20–1.83)	2.44 (1.74–4.37)
Third administration (day 3)	0.52 (0.35–0.79)	1.55 (1.17–1.89)	2.52 (1.74–4.38)
Fourth administration (day 8)	0.46 (0.30–0.68)	1.51 (1.11–1.93)	2.47 (1.75–3.68)
Fifth administration (day 9)	0.48 (0.34–0.75)	1.49 (1.10–1.98)	2.54 (1.68–3.94)
Sixth administration (day 10)	0.47 (0.25–0.66)	1.50 (0.91–2.24)	2.46 (1.13–3.92)
Over the six administrations	0.50 (0.25–0.85)	1.50 (0.91–2.24)	2.50 (1.13–4.38)

### Records

Hens were observed twice daily for general health throughout the study (last day: scheduled necropsy on day 11 or day 29 for hens selected for necropsy, or day 36). Physical examinations were performed by a masked veterinarian for all hens present on days -2, 4, 11, 29 and 36. These examinations included assessments of abnormalities in general appearance and behaviour, musculoskeletal system (e.g. locomotion, deformities), respiratory system (e.g. oculo-nasal discharge, abnormal breathing), integumentary system (e.g. abnormal feathering). In addition, a withdrawal reflex test was performed on each hen to test sensory and motor function in response to light pressure to the foot of each animal.

The individual food and water consumption were recorded daily from day -7 to day 35, and body weights were recorded regularly during the study (on assignment to the study and on days -16, -8, -1, 4, 7, 11, 18, 25, 29 and 36). Blood samples were collected for clinical pathology (hematology and clinical chemistry; Table 3) from birds allocated to blood sampling before the first treatment on day -3 and on days 4, 11, 21 and 29.

**Table 3:** List of clinical pathology parameters analyzed

Haematology	Clinical chemistry
Total white blood cell count (WBC)	Albumin
Differential WBC: Heterophils	Aspartate aminotransferase
Differential WBC: Lymphocytes	Calcium
Differential WBC: Monocytes	Chloride
Differential WBC: Eosinophils	Glutamate dehydrogenase
Differential WBC: Basophils	Creatine phosphokinase
Red blood cell count	Glucose
Platelets	Phosphate
Haematocrit	Potassium
Mean corpuscular volume	Sodium
Mean corpuscular haemoglobin	Total protein
Mean corpuscular haemoglobin concentration	Uric acid
Haemoglobin	Globulin (calculated as Total protein-Albumin)

Since hens were fed ad libitum during the present study, the determination of serum total bile acid concentrations would have been of limited diagnostic value and was therefore omitted. In addition, neither lactate dehydrogenase (LDH) nor gamma-glutamyl transferase (GGT) were determined from blood samples. Indeed, LDH isoenzymes are found in most avian tissues and an increased LDH activity has a low specificity for liver disease in avian species [10]. GGT is not considered a sensitive test for the detection of liver disease in avian species. Finally, no coagulation parameters were determined in the present study, either because of the lack of availability of appropriate measurement tools (prothrombin time and fibrinogen) or because of no relevance in this species (activated partial prothrombin time). However, the clinical pathology parameters investigated (e.g. determination of thrombocyte and red blood cell counts and haemoglobin values in the peripheral blood) and the post-mortem examination of the birds were appropriate to detect clinically relevant impairment of haemostasis.

From randomization to day 36, all the eggs laid by the 10 selected hens per treatment group were collected, recorded and subjected to evaluation of the following parameters: visual inspection for soundness of the shell and egg shape, egg shape (calculating the egg height to width ratio), egg shell thickness, egg strength, egg weight, albumen height, yolk color, Haugh unit (measure of the albumen quality as a function of albumen height and egg weight as defined by poultry industry [11]), presence of blood in egg, presence of meat spots on yolk.

To complete the safety assessment, the hens selected for necropsy underwent a post mortem examination, as required by VICH GL 43 [7]. On day 11 or day 29, hens were euthanized by cervical dislocation of the neck. A complete post-mortem examination was performed on all necropsied hens under the supervision of a blinded veterinary pathologist. Selected organs were weighed and multiple tissues were examined histopathologically (Table 4). Any gross lesion observed was collected and examined histopathologically as well. Tissue samples were formalin-fixed except the eyes were fixed in Davidson’s fixative. Microscopy slides were stained with hematoxylin and eosin stain. Tibiotarus bone marrow smears were prepared and stained with May Grunewald’s Giemsa stain. All samples from the control and from the 5× groups were assessed by a veterinary histopathologist.

**Table 4:** List of organs and tissues examined histopathologically and of organs weighed

Organ/tissue examined histopathologically	
Brain^a^	Tongue
Heart^a^	Oesophagus
Liver^a^	Crop
Spleen^a^	Proventriculus (stomach)
Thymus gland^a^	Ventriculus (gizzard)
Pituitary gland	Duodenum, jejunum, ileum, caecum
Thyroid and parathyroid glands	Bone (femur with marrow)
Adrenal glands	Marrow smear (from tibiotarus)
Pancreas	Bursa of Fabricius
Ovaries	Gall bladder
Uterus	Kidneys
Spinal cord (thoracic)	Cloaca
Peripheral nerves (sciatic nerve)	Colon
Eyes with optic nerve	Skeletal muscle (breast)
Larynx, trachea	Skin (breast)
Lung	Stifle joint

^a^Organ weighed

All the hens maintained beyond day 29 (until day 36) were euthanized on day 36 but were not subjected to gross necropsy and tissue collection.

### Analysis of the results

Body weight, food and water consumption, egg parameters (egg production, egg shell thickness, egg strength, egg weight and Haugh unit), and clinical pathology parameters collected during the main phase and the recovery phase 1 were statistically compared between groups (SAS® Version 8.2, SAS Institute Inc., Cary, NC, USA) using a repeated measures analysis of covariance with the individual hen as the experimental unit, and pre-treatment values as covariate, to evaluate the hypothesis that there are no differences between the groups. For data collected during the recovery phase 2, a one way analysis of variance was run. All the tests were performed at the 10% significance level. During the main study phase, in case of significant time*treatment group interaction, pairwise comparisons between each fluralaner-treated group and control group were performed at each timepoint. In case of non-significant time*treatment group interaction but significant treatment effect, pairwise comparisons between each fluralaner-treated group and control group were performed using linear contrasts. During recovery phases, in case of significant treatment effect, pairwise comparisons between each fluralaner-treated group and control group were performed using linear contrasts.

For clinical pathology parameters study-specific reference ranges were compiled, as these values were considered most suitable for the hen population examined. These reference ranges included results from the control group at all collection time points (before the onset of treatments on day -3, and on days 4, 11, 21 and 29) and from the fluralaner-treated groups on day -3. In support, historical data from healthy untreated laying hens of a similar strain and age was used to assess results. All clinical pathology parameters found to be statistically significantly different were compared with the study-specific reference ranges to evaluate the clinical relevance. Clinical relevance was assessed by the veterinary investigator based on the following criteria: transience (temporary observation), dose-response relationship, values close to or within the reference ranges, association with evidence of clinical signs and with tissue changes on gross post mortem or histopathological examination.

The veterinary investigator assessed all recorded parameters and any findings for their relationship to fluralaner treatment. Any clinically relevant treatment-related findings were classified as adverse events.

## Results and discussion

During the present study, the birds were applied a large overdosage with fluralaner, up to 5 times the recommended daily dose for 3 times the number of treatment days, compared to the recommended posology. Although the selected birds were considered under high physiological stress related to high egg production, no treatment-related findings were reported in any of the extensive list of parameters assessed, showing a wide safety margin for such a treatment compared with the expected field use conditions.

All the hens remained in good health for the duration of the experiment until their scheduled sacrifice time point. No abnormalities were detected for any hens at any of the veterinary clinical observations performed on days 4, 11, 29 and 36. There were no clinical findings related to treatment.

No change in water intake was detected on any of the 6 treatment days in any of the groups, and no statistically significant difference was shown between groups treated with medicated water and control group receiving unmedicated drinking water over the entire study period (Table 5). This shows that the addition of fluralaner, formulated as a 10 mg/ml solution, into drinking water at up to 5 times the recommended treatment dose, does not alter the acceptability of water by hens, which is of importance since reduced water intake may reduce egg production and live weight in laying hens [12]. There was no clinically relevant effect of treatment on food consumption by hens, except from an incidental higher food intake in 1× and 3× groups, but not in 5× group, during the recovery phase 1 (Table 5).

**Table 5:** Water and food intake (kg/day/bird) of hens, as mean^a^ per group, over the study

Group	Evaluation time (study period)			
	Pre-test -5 to - 1	Main study 1 to 10	Recovery1 11 to 28	Recovery2 29 to 34
Water intake				
Control	0.216	0.214	0.219	0.235
Fluralaner 1×	0.217	0.215	0.228	0.234
Fluralaner 3×	0.211	0.214	0.213	0.231
Fluralaner 5×	0.219	0.210	0.230	0.257
Food intake				
Control	0.111	0.111	0.104	0.103
Fluralaner 1×	0.115	0.112	0.111^b^	0.108
Fluralaner 3×	0.112	0.114	0.108^b^	0.107
Fluralaner 5×	0.110	0.111	0.107	0.107

^a^For post-treatment data, adjusted mean from the statistical model

^b^Statistically significantly higher food intake in 1× and 3× groups *vs* control from pairwise comparisons at each study period (*P* = 0.008 and *P* = 0.094, respectively)

Regarding body weight changes over the study period (Table 6), a significant time*treatment group interaction was detected during the main study phase (*P* = 0.084), with mean body weight in the 3× group higher than in controls on days 7 and 11. This increase was considered as incidental and not related to treatment with fluralaner as an increase in body weight was not observed in the higher dose (5×) group.

**Table 6:** Body weights (kg) of hens, as mean^a^ per group, over the study

Group	Evaluation time (study day)					
	Main study				Recovery1	Recovery2
	-1	4	7	11	18–25-29	36
Control	1.767	1.776	1.763	1.774	1.766	1.753
Fluralaner 1×	1.780	1.774	1.773	1.772	1.833	1.790
Fluralaner 3×	1.768	1.782	1.783^b^	1.795^b^	1.788	1.786
Fluralaner 5×	1.758	1.767	1.774	1.766	1.767	1.756

^a^For post-treatment data, adjusted mean from the statistical model

^b^Statistically significantly higher body weight in 3× group *vs* control from pairwise comparisons at each timepoint (*P* = 0.015 on day 7 and *P* = 0.063 on day 11)

There were no statistically significant differences between groups for egg production (the mean daily egg production per group was 1 egg/day in each group during each study phase), and the number of abnormal eggs, including soft shell eggs, abnormally large or small eggs, and broken/cracked eggs was comparable between the groups (1 egg in the 3× group, and 2 eggs in each of the other 0×, 1× and 5× groups over the 36-day period after the onset of treatment). Any hen that produced an abnormal egg was subject to additional health observations until 3 consecutive normal eggs had been laid. No clinical abnormalities were then observed in any of these animals. Treatment with fluralaner had no effect on the incidence of the presence of blood or meat spots in eggs. All the characteristics of eggs evaluated (as listed previously) were not significantly different between groups over the whole study. In particular, the quality of the albumen and egg protein content, as reflected by the Haugh unit value, was not influenced by treatment with fluralaner in any of the groups (Table 7).

**Table 7:** Egg quality parameters of eggs laid by hens, as mean^a^ per group, over the study

Group	Evaluation time (study period)			
	Pre-test -5 to -1	Main study 1 to 10	Recovery1 11 to 28	Recovery2 29 to 34
Egg weight (g)				
Control	58.4	58.9	58.0	59.0
Fluralaner 1×	59.0	59.5	59.8	59.9
Fluralaner 3×	58.7	59.3	60.0	60.6
Fluralaner 5×	58.8	59.1	60.0	60.7
Eggshell thickness (mm)				
Control	0.35	0.34	0.37	0.38
Fluralaner 1×	0.36	0.35	0.36	0.38
Fluralaner 3×	0.33	0.35	0.36	0.38
Fluralaner 5×	0.36	0.35	0.36	0.37
Eggshell strength (kg.f)				
Control	4.860	5.268	5.032	5.005
Fluralaner 1×	5.332	4.992	5.255	5.233
Fluralaner 3×	5.061	5.238	5.141	5.113
Fluralaner 5×	5.211	5.087	5.151	5.025
Egg haugh unit				
Control	80.2	79.4	77.0	78.1
Fluralaner 1×	82.1	80.2	76.4	73.5
Fluralaner 3×	79.0	80.3	77.5	75.0
Fluralaner 5×	80.4	79.4	76.2	75.1

^a^for post-treatment data, adjusted mean from the statistical model

Regarding clinical pathology, statistically significant differences between treated and control birds were found for 2 hematology parameters (monocytes and basophils) and 6 clinical chemistry parameters (calcium, phosphate, uric acid, glutamate dehydrogenase, albumin and total protein). During the main phase, the treatment effect was statistically significant for calcium, with a higher adjusted mean in the 5× group, and the treatment group*time interaction was significant for phosphate, with no difference between control and treated groups at any time point. During the recovery phase 1, the treatment effect was statistically significant for monocytes (higher adjusted means in the 1× and 3× groups), glutamate dehydrogenase (higher adjusted mean in the 5× group) and for basophils, with no difference between the control group and any treated group overall. The treatment group*time interaction was significant for uric acid, total protein and albumin, with no difference between the control and treated groups at any timepoint (uric acid and total protein) or with a lower adjusted mean in the 5× group on day 29 (albumin). None of these findings were considered to be of clinical relevance. The statistically significant differences observed between the control and fluralaner-treated groups were deemed to be either minimal, with values remaining within the range of values reported in untreated hens, lacking clear dose relationships, or of no biological relevance and were therefore considered not related to treatment. In addition, there were no clinical, gross or histopathology findings which correlated with the differences observed.

There were very few isolated necropsy findings across all treatment groups (5 and 3 gross findings out of 32 birds necropsied per timepoint, on days 11 and 29, respectively), all being limited to one finding per bird. The observations included dark fluid accumulation in the abdominal cavity, pale liver discoloration, dark red discoloration of left thyroid gland, dark red discoloration of left parathyroid gland, and abnormal appearance of the uterus/persistent right oviduct, dark red discoloration of the bursa of Fabricius and presence of a cyst on the bursa of Fabricius. None of these findings were correlated with any pathological process at histology evaluation and they were observed in isolated animals. They were considered as incidental, of a nature commonly observed in this strain and age of laying hens, and/or of similar incidence in control and fluralaner-treated groups. All organs from the treated groups had a weight comparable with the control group, except thymus which was reported lighter in fluralaner-treatment groups. A large variation in thymus weights is not unexpected in this class of chickens as the thymus starts to regress at sexual maturity [13], and thymus glands are thus difficult to collect accurately and are of small mass (mean weight below 1–1.5 g). Furthermore, there were no gross or histological findings for the thymus glands, and there was no direct correlation between size of the thymus and dose level and no noticeable weight difference and/or gross or histopathological findings in the other primary lymphoid organs (spleen and bursa of Fabricius). Thus, the thymus weight finding was considered related to normal variation in the involution of the thymus at sexual maturity and not related to the administration of fluralaner. The histopathological examinations conducted in control and 5× groups did not reveal any findings associated with fluralaner administration.

## Conclusions

This detailed evaluation of the safety of fluralaner, a novel systemic acaricide for poultry mite treatment in layers, following oral administration at doses much higher than the recommended treatment dose and at a shorter interval, did not reveal any treatment-related adverse events in laying hens and on their egg production. Oral administration of fluralaner, administered *via* drinking water to laying hens at dose rates of up to 2.5 mg/kg on 6 occasions did not lead to any treatment-related findings that could be detected through careful clinical observations, egg production and quality assessments, clinical pathological evaluation or gross or microscopic post mortem examination. Oral administration of fluralaner at the recommended treatment dose (twice 0.5 mg/kg at 7 days interval) is well tolerated by laying hens, with a high safety margin up to an overall dose of 15 times the recommended one (5 times the daily dose given 3 times the number of days). Based on the present results, the use of the new mite treatment based on fluralaner administered *via* drinking water is expected to be safe for laying hens under industrial conditions, and to have no negative impact on their egg quality and production.

## Abbreviations

GABA: γ-aminobutyric acid
GGT: Gamma-glutamyl transferase
GLP: Good Laboratory Practice
LC: Liquid chromatography
LDH: Lactate dehydrogenase
UV: Ultraviolet
WBC: White blood cell count
